# Supplementary material for: Intensive care unit sinks are persistently colonized with multidrug resistant bacteria and mobilizable, resistance-conferring plasmids
Source: mSystems. 2023 Jul 13;8(4):e00206-23. doi: 10.1128/msystems.00206-23 (PMC10469867; doi:10.1128/msystems.00206-23)
Supplement: Table S1 — Individual plasmids. [file msystems.00206-23-s0008.docx]

| Cluster | Mean Length (bp) | Mean GC % | Replicon type(s) | Relaxase type | MPF type | ARG(s) | Species in cluster |
| --- | --- | --- | --- | --- | --- | --- | --- |
| 1 | 67328.2 | 53.3 | IncN | MOBF | MPF_T | aac(6')-Ib-cr5, aadA16, arr-3, blaNDM-5, blaTEM-1, ble, dfrA27, mph(A), qnrB6, sul1, tet(A) | *K. pneumoniae, C. freundii, K. michiganensis, E. hormaechei* |
| 2 | 6141.0 | 52.2 | rep_cluster_1195 | MOBP | - | blaOXA-232 | *K. pneumoniae* |
| 3 | 177388.5 | 54.4 | - | - | - | aadA1, blaNDM-1, blaOXA, blaPER-3, ble, mph(A), sul1 | *A. caviae* |
| 4 | 110792.0 | 54.8 | IncFIB,IncFII,rep_cluster_2272 | MOBF | MPF_F | blaNDM-1, ble, rmtC, sul1 | *C. freundii* |
| 5 | 309511.3 | 48.8 | Col(VCM04) | MOBH | - | aac(3)-IIe, aac(6')-Ib-cr5, aadA1, aadA16, arr-3, blaCTX-M-15, blaNDM-1, blaOXA-1, ble, catA2, dfrA27, sul1, sul2 | *C. freundii* |
| 6 | 222581.3 | 55.1 | rep_cluster_1332 | MOBH | - | aac(3)-IId, aac(6')-Ib, aac(6')-Ib-cr5, aadA1, aadA16, ant(3'')-Ij/aac(6')-Ib, aph(3'')-Ib, aph(6)-Id, arr-3, blaNDM-1, blaOXA, blaOXA-1, blaPER-3, blaTEM-1, ble, catB3, mph(A), sul1, tet(A) | *A. caviae* |
| 7 | 85098.0 | 57.2 | - | - | - | aac(6')-Ib4, aac(6')-Il, aadA5, arr-2, blaIMP-1, blaOXA-10, dfrA15, sul1, tmexC3, tmexD3, toprJ1 | *P. stutzeri* |
| 8 | 329536.5 | 47.1 | IncHI2A,rep_cluster_1088 | MOBH | MPF_F | aac(6')-Ib-cr5, aadA1, aadA16, arr-3, blaNDM-1, blaOXA-1, ble, catA1, dfrA27, qnrB1, sul1, tet(A) | *E. coli, C. freundii* |
| 9 | 333080.0 | 47.1 | IncHI2A,rep_cluster_1088 | MOBH | MPF_F | aac(3)-IIe, aac(6')-Ib-cr5, aadA1, aadA16, arr-3, blaNDM-1, blaOXA-1, blaTEM-1, ble, catA1, dfrA27, mph(A), mph(E), msr(E), qnrB1, sul1, tet(A) | *E. roggenkampii, E. hormaechei* |
| 10 | 331845.0 | 38.8 | - | MOBP,MOBP | - | aac(6')-Ib4, aph(3'')-Ib, aph(3')-VIa, aph(6)-Id, arr-3, blaNDM-1, blaOXA-58, ble, dfrA44, mph(E), msr(E), sul1, sul2, tet(X3) | *A. junii, A. johnsonii* |
| 11 | 302786.5 | 39.3 | - | MOBP | - | aac(6')-Ib4, aph(3'')-Ib, aph(3')-VIa, aph(6)-Id, arr-3, blaNDM-1, ble, mph(E), msr(E), sul1, sul2, tet(X3) | *A. johnsonii* |

**Table 1** Summary of plasmid clusters
