# Supplementary material for: Intensive care unit sinks are persistently colonized with multidrug resistant bacteria and mobilizable, resistance-conferring plasmids
Source: mSystems. 2023 Jul 13;8(4):e00206-23. doi: 10.1128/msystems.00206-23 (PMC10469867; doi:10.1128/msystems.00206-23)
Supplement: Supplemental Material Legends — Legends for Fig. S1 to S7, Table S1, and Data Set S1. [file msystems.00206-23-s0009.docx]

**Supplementary Material**

**Supplementary Figures**

**Supplementary Figure 1** Detailed breakdown of recovered isolates **(A)** Genera of recovered isolates in this study **(B)** Species of recovered isolates in this study **(C)** Species, annotated by country of collection **(D)** Species, annotated by site of collection (ICU vs. Home/Work)

**Supplementary Figure 2** **(A,C,E,G,H,J,L)** Maximum likelihood phylogenetic tree *of 7 different taxa*, generated from individual core genome alignment. Metadata is colored as rings (from inside out): Strain, Study Month, Room class, and Surface. **(B,D,F,I,K,M)** Space and time information for isolates. Shapes represent at least one isolate was recovered at that time point, and rows represent different rooms. Shapes colored by strain identity.

**Supplementary Figure 3 (A-B)** Stacked bar plots showing absolute number of isolates collected from each species in US/PAK, colored by room class. Bars to the left represent genera found more in ICUs, and bars to the right represent genera found more in HOME/WORK rooms. Each bar is annotated with heatmaps showing the mean number of ARGs (red/orange) and ARG classes (green/blue) in each genus. *** = P<0.001, ** = P<0.01, * = P<0.05 by pairwise Wilcoxon tests with Benjamini-Hochberg adjustment. Individual group sizes and ARG counts are noted in Supplementary Data 1

**Supplementary Figure 4** Maximum likelihood phylogenetic tree of *P. stutzeri* isolates, generated from core genome alignment. Annotations indicate the strain identity, month of collection, absolute ARG count, and identity of specific beta-lactamases that were annotated in each genome. Note that strain 3 isolates (light blue) were collected over 25 months apart and harbor *bla*_VIM-2_ in their genome, demonstrating strain persistence as a mechanism for ARG persistence in this environment

**Supplementary Figure 5** **(A)** Nucleotide alignment of shared cluster 1 plasmids. Grey blocks show BLAST matches of >99% ID and > 5 kb, with SNV counts on the left, and the ANI (based on SNVs) noted on the right. ORFs are colored by function (pink = ARG, orange = MGE, teal = other). The AST results of the bolded isolate is displayed in the panel B **(B)** AST interpretations of all isolates from all strain 1 *K. michiganensis* isolates, with beta-lactamase annotations on the right. Hybrid assembly of PK1-MO2-ICU-D4 demonstrated presence of the circularized plasmid sequence displayed in panel A. Another isolate, PK1-MO2-ICU-B2, did not possess the ARGs harbored on the sequenced plasmid elsewhere in its genome and was susceptible to most of the beta-lactamases tested, indicating that presence of this plasmid is sufficient for resistance to these antibiotics.

**Supplementary Figure 6** Histogram of ANI values based on SNVs calculated in an all-vs-all manner within species. The line indicates where the cutoff was placed for the definition of strain in this study

**Supplementary Figure 7** Principal coordinate analysis of *P. aeruginosa* accessory genome. The accessory genomes of individual *P. aeruginosa* strains were significantly different from one another by Jaccard distance **(A-B)** (P < 0.001, PERMANOVA), and strains that were recovered at multiple time points had significantly different accessory genomes compared to strains that were only recovered from a single time point (P < 0.001, PERMANOVA) **(C-D)**

**Supplementary Tables**

**Supplementary Table 1** Individual plasmid types and metadata

**Supplementary Data**

**Sheet 1:** Isolate metadata

**Sheet 2:** Strain metadata and SNP counts

**Sheet 3:** Isolate and ARG count by room class + country

**Sheet 4:** ARG counts by genus

**Sheet 5:** Wilcoxon results (ARG count by genus)

**Sheet 6:** ARG counts by species

**Sheet 7:** Wilcoxon results (ARG count by species)

**Sheet 8:** AST results
